# Take home injectable opioids for opioid use disorder during and after the COVID-19 Pandemic is in urgent need: a case study

**DOI:** 10.1186/s13011-021-00358-x

**Published:** 2021-03-05

**Authors:** Eugenia Oviedo-Joekes, Scott MacDonald, Charles Boissonneault, Kelli Harper

**Affiliations:** 1 School of Population and Public Health, University of British Columbia, 2206 East Mall, Vancouver, BC V6T 1Z3 Canada; 2 Centre for Health Evaluation & Outcome Sciences, Providence Health Care, St. Paul’s Hospital, 575- 1081 Burrard St, Vancouver, BC V6Z 1Y6 Canada; 3 Providence Health Care, Providence Crosstown Clinic, 84 West Hastings Street, Vancouver, BC V6B 1G6 Canada; 4 British Columbia Centre on Substance Use, BC Centre on Substance Use (BCCSU), 400-1045 Howe St, Vancouver, BC V6Z 2A9 Canada

**Keywords:** Opioid use disorder, Patient centered care, Injectable opioid agonist treatment, Diacetylmorphine, Take home doses, Direct observed treatment

## Abstract

**Background:**

In North America the opioid poisoning crisis currently faces the unprecedented challenges brought by the COVID-19 pandemic, further straining people and communities already facing structural and individual vulnerabilities. People with opioid use disorder (OUD) are facing unique challenges in response to COVID-19, such as not being able to adopt best practices (e.g., physical distancing) if they’re financially insecure or living in shelters (or homeless). They also have other medical conditions that make them more likely to be immunocompromised and at risk of developing COVID-19. In response to the COVID-19 public health emergency, national and provincial regulatory bodies introduced guidance and exemptions to mitigate the spread of the virus. Among them, clinical guidance for prescribers were issued to allow take home opioid medications for opioid agonist treatment (OAT). Take Home for injectable opioid agonist treatment (iOAT) is only considered within a restrictive regulatory structure, specific to the pandemic. Nevertheless, this risk mitigation guidance allowed carries, mostly daily dispensed, to a population that would not have access to it prior to the pandemic. In this case it is presented and discussed that if a carry was possible during the pandemic, then the carry could continue post COVID-19 to address a gap in our approach to individualize care for people with OUD receiving iOAT.

**Case presentation:**

Here we present the first case of a patient in Canada with long-term OUD that received take home injectable diacetylmorphine to self-isolate in an approved site after being diagnosed with COVID-19 during a visit to the emergency room where he was diagnosed with cellulitis and admitted to receive antibiotics.

**Conclusion:**

In the present case we demonstrated that it is feasible to provide iOAT outside the community clinic with no apparent negative consequences. Improving upon and making permanent these recently introduced risk mitigating guidance during COVID-19, have the potential not just to protect during the pandemic, but also to address long-overdue barriers to access evidence-based care in addiction treatment.

## Background

In North America the opioid poisoning crisis currently faces the unprecedented challenges brought by the COVID-19 pandemic, further straining people and communities already facing structural and individual vulnerabilities. For example, in the United States, opioids are involved in at least two thirds of the 128 apparent overdose deaths reported per minute [1]. In Canada, and similar to the US, after a slight decline in 2019, a rise in apparent overdose deaths is been seen in 2020 with the advent of the COVID-19 pandemic [2, 3].

Opioid agonist treatment (OAT) with oral long acting medications is the most widely used approach for opioid use disorder (OUD) in Western countries, with proven effectiveness. Most patients will stop or reduce their use of street opioids, and may improve their physical and mental health and social connections [4]. Another effective alternative treatment available in some European and Canadian settings, is injectable OAT (iOAT) with either diacetylmorphine or hydromorphone [5, 6].

The delivery of iOAT comes with significant more restrictive regulatory limits compared to oral OAT. One of the main premises of the iOAT provision is that the medications are dispensed and self-administered by injection under direct observation, for the safety of the patient and the community [7, 8]. When the medications are taken onsite, patients can be monitored for signs of intoxication before the injection or after, for signs of over-sedation or respiratory depression. Moreover, if a dose intolerance (i.e., overdose) occurs after the injection of the medication, the observation allows for immediate onsite treatment, ensuring the safety of the patient [9]. The risk for the community stems by the possibility of diversion of the patients’ medication [10], or its use not as prescribed, posing risk to others. Take home doses [7, 11] (also called carries) for injectable medications are not allowed in this framework, even if clinically advised.

Under a different path, and currently to a different and limited patient profile [11], the United Kingdom offers injectable diacetylmorphine (and methadone) as carries in conditions that would mirror what guidelines recommend when assessing clinically the provision of take home doses of OAT with methadone or buprenorphine. The assessment for take home doses is left to the prescriber [11, 12], and recommendations are made regarding the safety of the community (e.g., safe storage, diversion, etc.), management of potential adverse events and overall patient stability (e.g., other substance use problems that can interfere with the medication dispensation). Although this approach is in decline and has not expanded to any other context worldwide [13] it is noteworthy that until the ‘90s carries were the only way to access diacetylmorphine in the UK [14].

In response to COVID-19, people with OUD are facing unique challenges, such as not being able to adopt best practices (e.g., physical distancing) if they’re financially insecure or living in shelters (or homeless). They also have other medical conditions that make them more likely to be immunocompromised and at risk of developing COVID-19. For example, provincial reports show that a higher proportion of people who had a non-fatal overdose had chronic pulmonary disease, coronary heart disease, and multiple chronic health conditions [15]. Thus, people already in vulnerable situations are been made even more vulnerable in a pandemic.

In response to the COVID-19 public health emergency, national and provincial regulatory bodies introduced guidance and exemptions to mitigate the spread of the virus in the community. In order to facilitate patients to self-isolate, clinical guidance for prescribers were issued to allow or expand carries for oral OAT, such as slow release oral morphine (SROM) and immediate release hydromorphone tablets, [16, 17]. Take home of iOAT were included in these documents, however with very restrictive regulations and only when self-isolating in approved sites. Nevertheless, the intersection of these two public health emergencies brought a revision of practices and as a result a set of risk mitigation guidance regarding carries. These guidance aim at keeping the patient and the community safe of COVID-19 community transmissions but also to make sure there is continuation of care for OUD patients regarding their opioid medication. Switzerland has revised such policies for iOAT carries restrictions with diacetylmorphine until December 2021 [18].

The COVID-19 epidemic is disproportionately adversely affecting people who use illicit opioids and other street drugs but it is also leading to opportunities to improving treatment delivery. iOAT has been proven to be effective through several randomized controlled trials (RCT) [19, 20], and its continuation and expansion beyond the protocols and context of the RCTs requires a careful revision to ensure it is optimal, not just from the public health safety perspective but also from a patient-centered care approach. Here we present the first case of a patient in Canada with long-term OUD that received take home injectable diacetylmorphine to self-isolate in an approved site after being diagnosed with COVID-19 during a visit to the emergency room where he was diagnosed with cellulitis and admitted to receive antibiotics. This risk mitigation guidance allowed carries, mostly daily dispensed, to a population that would not have access to it prior to the pandemic. In this case it is presented and discussed that if a carry was possible during the COVID-19 pandemic, then the carry could continue post COVID-19 to address a gap in our approach to individualize care for people with OUD receiving iOAT.

## Case presentation

A 48 years old male, with a history of opioid use disorder (OUD) and 33 years of injection opioid use, was receiving iOAT with diacetylmorphine (DiaMo) at a community clinic in Vancouver for 6 years. Prior to accessing this treatment, he tried oral methadone 5 to 6 times up to 280 mg, which was not well tolerated due to side effects and led to continue to use street heroin. He also attempted residential and 12 step programs. Prior to iOAT he had overdoses requiring emergency services and administration of naloxone and had involvement with the criminal justice. He was being prescribed injectable diacetylmorphine 400 mg twice a day and 400 mg daily of slow release oral morphine (SROM, Morphine Sulfate 200 Mg Capsules 12 Hour Pellet M-Eslon Ethypharm Inc.), the latter if needed. Over the course of his treatment with iOAT, he never had a dose intolerance with either medication. The patient has several medical comorbidities: Infective endocarditis in 2009 with aortic root and mitral valve replacement, Gastroesophageal Reflux Disease and esophagitis, prior pulmonary embolism, stimulant use disorder (methamphetamine), tobacco use disorder, sensorineural hearing loss. He lives alone in a single room occupancy (SRO) hotel, a low income housing in the Downtown East Side Vancouver area.

On 21 September, on his own accord, he attended St. Paul’s Hospital emergency room, with a painful swollen right leg and fever. He was diagnosed with cellulitis, given intravenous (IV) antibiotics, and discharged. As a routine, and due to the flu-like symptoms, a nasopharyngeal swab was done for COVID-19. During the screening, he reported not being aware of having any contacts with COVID-19 positive individuals. He was referred to the outpatient parenteral antibiotic program (OPAT) for more IV antibiotics the next day and he was then discharged. That evening, he attended the iOAT community clinic to receive his medications, wearing a mask.

On 22 September he spoke on the phone with the infectious disease doctor at OPAT who noted the COVID-19 Coronavirus (PCR/NAAT) test result was positive from the previous day and asked the patient to go the hospital. Upon receiving his COVID-19 diagnosis, the patient reported to be scared and went to the iOAT clinic to share the news and for support. He did not want to come in because he knew of his positive test (he was concerned for the safety of the people inside), but he asked for help on how to get to the hospital. He was received at the door with personal protective equipment and given reassurances. From there, he was assisted to get a taxi, and went back to St. Paul’s emergency room. While his COVID-19 could have been managed in the community, he was admitted in the hospital for management of his cellulitis and IV antibiotics. Vice versa, his cellulites could have been managed in the OPAT, typically in 2 days, but due to the COVID-19 diagnosis, they could not isolate him there.

During the hospitalization, and in order to continue with his OUD treatment, the hospital team offered the patient alternative OAT, since diacetylmorphine in Canada is not available in acute care. To date, St. Paul’s Hospital rules do not allow for patients to self administer IV opioid medications for OUD, contrary to how it is done in the iOAT community clinic, where assistance is only provided for IM (intramuscular) doses, if requested. For patients on iOAT admitted to the hospital, the usual protocols involve administration of intravenous hydromorphone by nursing staff, typically combined with an oral long-acting opioid. Both agents are titrated to cover opioid withdrawal and cravings. The patient declined injectable hydromorphone, reporting he does not find it potent enough and precipitates withdrawal symptoms. To replace his diacetylmorphine and meet his opioid needs, the addiction medicine consultation team provided him with intravenous fentanyl and, because the duration of action of IV fentanyl is typically short, fentanyl patches were added to the patient’s usual SROM dose (see Table 1).

**Table 1:** Nursing Medication Administration Record (MAR)

Medication	Fentanyl patch	SROM (MEslon©)	IV Fentanyl	Sufentanil	Morphine (PO, IR)
Route of administration	Transdermal patch	Oral	Intravenous PRN	Sublingual PRN	Oral PRN
September 22nd	200 mcg/h	400 mg daily			4 doses of 50 mg po
September 23rd	250 mcg/h	400 mg daily	2 doses of 1000 mcg		4 doses of 50 mg po
September 24rd	250 mcg/h	400 mg bid	2 doses of 1000 mcg	1 dose of 200 mcg	
September 25th	250 mcg/h	400 mg bid	3 doses of 1000 mcg	1 dose of 200 mcg 1 dose of 250 mcg	1 dose of 50 mg 1 dose of 300 mg
September 26th	250 mcg/h	400 mg bid	7 doses of 1000 mcg		
September 27th	250 mcg/h	400 mg bid	7 doses of 1000 mcg		
September 28th	250 mcg/h	400 mg daily	7 doses of 1000 mcg		

*IR *immediate release, *PRN* Administered on an as-needed basis, *mcg/h* Microgram per hour, *mg* Milligram, *mcg* Microgram, *bid* twice per day, *PO* Oral formulation

Because IV access was not initially available and the treating team was worried the patient might leave the hospital to meet his needs with street opioids, sublingual sufentanil and immediate release oral morphine were also prescribed to allow for a more rapid titration. Once IV access was obtained, IV fentanyl was provided in 1000 mcg increments on an as-needed basis, every 2 hours PRN initially, then every hour as the patient was still reporting withdrawal symptoms with slower titration. Monday September 28th, he was discharged to a transitioning housing, a hotel leased by the health authorities to support people who need to isolate with COVID-19, with staff support, health care providers and meals.

During the patient’s hospitalization (on September 24th), the iOAT community clinic team was contacted by the hospital team and planning was started for his eventual discharge to the temporary housing, including provision of iOAT with diacetylmorphine and SROM (i.e., his regular OUD treatment prescription). Once discharged from the hospital and in the transitioning housing, his medications were delivered twice daily by a pharmacist and a nurse from his iOAT site, and the use of the injection diacetylmorphine witnessed. When the patient was not in his room, no dose was provided. He reported some evening withdrawal to the pharmacist so his SROM was changed to: SROM 400 mg (witnessed), another 400 mg (carry for later). He engaged well with the care team, and he was thankful to be receiving outreach care (he was proudly showing off his newly dyed hair), however he expressed it was unnecessary to come so many times per day (i.e., once would have been enough). In this particular case, it was helpful to have the nursing staff who know the client to join the visit as they know him and his baseline well. He reported strongly disliking the transitional place (i.e., not clean) he was in, and would have prefer to self-isolate in his own place and receive his medications there. He had some persistent chills, cough and runny nose and therefore his stay was extended at the transitioning housing for a few more days. He was cleared from isolation on October 13th and attended the community iOAT clinic to receive his regular doses.

## Discussion

Patient centered cared (PCC) has become a core principle in many medical fields, with health organizations, institutions and regulatory bodies acknowledging the importance of integrating the patients as partners in the medical decisions that directly impact their lives [21, 22]. Although it varies across disciplines and settings, the principles of PCC most commonly include [23] 1) shared power and responsibility between the patient and health care provider with shared decision-making (e.g., communication); 2) an individualized approach by focusing on the patients’ unique goals, needs and preferences with an understanding of the whole person; and 3) emphasis in the quality of the therapeutic relationship with the provider. Yet, addiction medicine struggles to incorporate these principles, particularly in the presence of prescription of opioid medications [24]. Honoring patients’ preferences in the medications they would choose to take, the setting and the format of the treatment are sometimes limited by the restrictive regulatory structures [25] (e.g. drug scheduling), unfavorable physical conditions (e.g. lack of housing), or lack of providers’ training in PCC in addictions (e.g., leading to stigmatizing care).

The rapid measures taken by the health care system in some settings to protect the public from the spread of COVID-19 has allowed patients with OUD to access treatment and approaches to care long-time needed, such us more flexibility with take home medications or carries. During the surge of COVID-19 several settings revised their policies, through provisional public health exemptions, allowing prescribers more flexibility prescribe OAT, including risk mitigation options and to offer carries [26, 27].

The global standard for iOAT (excluding the small group of patients receiving iOAT from pharmacies in the UK) is that is it excluded from any clinical guideline for take home “privileges” due to safety concerns (to the patient and diversion). This is blanket policy that does not allows for any shared decision making or consideration of the patient as a whole, including individual needs and preferences, directly contradicting the PCC values that medical science is committed to uphold.

In the present case, the patient received his 400 mg of injectable diacetylmorphine twice a day in temporary setting for self isolation due to COVID-19. At the end of his isolation period the patient went back to his SRO, clearly indicating (and confirmed by clinical assessment) that the optimal course of treatment for him would have been to receive carries that allowed less visits to the clinic, due to his lengthy prior medical conditions (e.g., physical challenges, fatigue). However, this was not possible due to restrictive regulatory structures, out of the control of provider (e.g., prescribe for take home) and the patient (e.g., setting to receive the medication). If providing iOAT carries was possible as a risk mitigating measure during COVID-19, this case shows that it is possible to consider carries for iOAT outside the scope of a COVID-19 infection case. While there are many other factors to be considered for carries, this presents an opportunity to consider carries for clients that have shown 1) willingness or record of adhering to the medication, as a way for the clinical team to assess that this is the formulation the person prefers and will continue using outside the premises; 2) evidence the patient will tolerated the dose for the time considered, and bearing in mind that there is constant consultations with the clinical team and shared-decision making if circumstances change (e.g., aggravation of COPD) and 3) patients needs and preferences regarding coming to clinic due to physical health, in this case, that makes it difficult to daily access the site where their trusted connections with the healthcare system are located.

One of the major factors driving the hospital team’s effort to quickly titrate opioids and to resort to IV fentanyl (since diacetylmorphine was not available in hospital), was the fear that the patient might self-initiate discharge from hospital to access street drugs, thereby breaking isolation and risk of overdose. To our knowledge, there is no previously published literature on the use of IV fentanyl in the context of hospital admission for patients with OUD. While this practice was used as a last resort in this specific case, providing as close of a pharmacologic agent to what patients are using in the street can be a way to support them staying engaged in acute treatment. This might allow to meet patient’s most immediate medical needs, whether those are related to COVID-19 self-isolation or any other emergency. While further research is certainly mandated before implementing more broadly such an approach, the flexibility and patient center care deployed here were crucial in keeping this patient engaged in the therapeutic alliance and allowed for collaboration in planning for a safe hospital discharge.

Over the years, the concern on what will happen with Scheduled I and II medications once they leave the premises have limited the quality of the care provided in addiction medicine, due to stigmatizing and over restricted regulations (mostly from the US politics that serve still as international standard) [28]. There are recognised negative associated consequences, when medications such as opioids are not used as directed (e.g., poor treatment outcomes, fatal overdoses, increase in crime, etc.) [29]. However, while most of the emphasis in research and policy has been around tougher measures of control (e.g., special licenses to prescribe, “deterrents”, etc.), or increased patient monitoring, it has come at the expense of patient autonomy [30]. Little is known (and little attention paid) to the motivations and factors that contribute to diversion at the patient level [31, 32]. Understanding the specific circumstances of the patient as a whole and addressing other needs (when possible) that could be interfering with the treatment and leading to the diversion of the medication would be a patient-centered strategy [10, 33]. These needs could be but not limited to physical and medical comorbidities, other substance use, partners or friends in need of medication, and overall financial stress.

Singling out iOAT from the option of carries introduces a lack of equity for a group of patients without considering their specific circumstances and needs, and responds only to restrictive policies. Moreover, allowing iOAT carries in such a restrictive way that those that really need it cannot access it, is also lack of equity (e.g., if carries can only be prescribed to people with stable housing). In the present case we demonstrated that it is feasible to provide iOAT outside the community clinic with no apparent negative consequences. While the dispensation was witnessed by the health care providers still, this will not be needed once the patient and providers are comfortable with the process, making it less expensive and time consuming. In Switzerland, currently due to COVID-19, patients are allowed to take seven daily doses of carries [18]. A first step in our context will be to work with the local authorities and include patients in iOAT that could benefit from carries, in the sense of optimizing their quality of care, without burdening the system with unnecessary monitoring, such us direct observed treatment and twice daily deliveries. Patients in iOAT come two to three times a day, and over time they report a strong need of less visits but maintain the connection with the clinic [34] (as in the present case). Allowing flexibility based on individual assessment anchored in PCC can support patients’ needs and quality of care.

To date, our community iOAT clinic has seen 7 cases of COVID-19, this been the first that received injectable diacetylmorphine in the transition housing during the isolation period. At the clinic, all staff wears medical grade masks and goggles. At that time, patients were encouraged to wear a mask, although now require all clients and staff members to wear medical grade masks while in clinic. Social distance within clinic is maintained and engagement is 100%. There have been no COVID-19 transmissions.

A public health emergency of this extent and complexity requires a comprehensive response that embraces innovation while exhausting evidence-based approaches. The overdose crisis, now aggravated by the COVID-19 pandemic as it rapidly evolves, makes this a critical time for iOAT normalization within the addiction treatment system. The street supply is growing even more unpredictable as normal supply channels are disrupted (e.g., difficulty finding sources due to social distancing), and supports for people who use drugs are strained. The failure to deliver effective treatment for opioid use disorder translates in loss of in-person treatment options. This case illustrates that the system is in a position to provide continuation of iOAT care to people in the midst of this new public health emergency, but also that we can do so after, as long as processes are in place to support the patient and the community, in a sustainable way. People with OUD struggle to adopt COVID-19 best practices (e.g., physical distancing) for diverse reasons (e.g., living in shelters, clinic visits). This can affect their safety and treatment progress by, for example, reducing their time spent in treatment, or increasing the sense of insecurity. As data shows, the COVID-19 epidemic has tremendously affected the incidence of opioid overdose but is bringing us opportunities to reduce overdoses by improving treatment and enhancing linkage to care.

## Conclusion

Guidelines to a more or less extent, allow for a medical assessment of the patients’ circumstances and treatment history to discuss the possibility and arrangement of the carries with oral OAT upon considering optimal outcomes for the patient and public health [12, 35]. However, iOAT has been exempt of these evidence-based approaches or made so restrictive that they cannot be applied to those who need them. In the present case we demonstrated that it is feasible to provide iOAT outside the community clinic with no apparent negative consequences. Improving upon and making permanent these recently introduced risk mitigating policies during COVID-19, have the potential not just to protect during the pandemic, but also to address long-overdue barriers to access evidence-based care in addiction treatment. If providing iOAT outside of the clinical settings is being successful now, thus, it can also be successful later, too.

## Data Availability

Data sharing is not applicable to this article as no datasets were generated or analysed during the current study.

## Abbreviations

OUD: Opioid use disorder
OAT: Opioid agonist treatment
iOAT: Injectable opioid agonist treatment
IV: Intravenous
OPAT: Outpatient parenteral antibiotic program
IM: Intramuscular
SROM: Slow release oral morphine
PCC: Patient centered cared

## References

[1] CDC (2020). Combatting the Opioid Overdose Epidemic.

[2] Service BC (2020). Illicit drug toxicity deaths in BC January 1, 2010 – September 30.

[3] O’Donnell J, Gladden RM, Mattson CL, Hunter CT, NL D. (2020). Vital signs: characteristics of drug overdose deaths involving opioids and stimulants: 24 states and the District of Columbia, January-June 2019. MMWR Morb Mortal Wkly Rep.

[4] Bell J, Strang J (2020). Medication treatment of opioid use disorder. Biol Psychiatry.

[5] Maghsoudi N, Bowles J, Werb D (2020). Expanding access to diacetylmorphine and hydromorphone for people who use opioids in Canada. Can J Public Health.

[6] Strang J, Groshkova T, Uchtenhagen A, van den Brink W, Haasen C, Schechter MT (2015). Heroin on trial: systematic review and meta-analysis of randomised trials of diamorphine-prescribing as treatment for refractory heroin addictiondagger. Br J Psychiatry.

[7] Strang J, Groshkova T, Metrebian N, Strang J, Groshkova T, Metrebian N (2012). New heroin-assisted treatment: recent evidence and current practices of supervised injectable heroin treatment in Europe and beyond: EMCDDA.

[8] Oviedo-Joekes E, Palis H, Guh D, Marsh DC, MacDonald S, Harrison S, Brissette S, Anis AH, Schechter MT. Adverse Events During Treatment Induction With Injectable Diacetylmorphine and Hydromorphone for Opioid Use Disorder. J Addict Med. 2019;13(5):354-61. 10.1097/ADM.0000000000000505 PMC6791495 30747750

[9] Oviedo-Joekes E, Brissette S, MacDonald S, Guh D, Marchand K, Jutha S (2017). Safety profile of injectable hydromorphone and diacetylmorphine for long-term severe opioid use disorder. Drug Alcohol Depend.

[10] Fountain J, Strang J, Gossop M, Farrell M, Griffiths P (2000). Diversion of prescribed drugs by drug users in treatment: analysis of the UK market and new data from London. Addiction.

[11] Department of Health (England) and the Chief Medical Officers of the UK (2017). Drug misuse and dependence.

[12] British Columbia Centre on Substance Use and B.C (2017). Ministry of Health.

[13] Mayet S, Manning V, Sheridan J, Best D, Strang J (2010). The virtual disappearance of injectable opioids for heroin addiction under the ‘British system’. Drugs Educ Prev Policy.

[14] Strang J, Gossop M (1996). Heroin prescribing in the British system: historical review. Eur Addict Res.

[15] Slaunwhite A, Gan W, Desai R, Zhao B, Xavier C, J. B (2020). Overdose and Risk Factors for Developing Severe Acute Respiratory Syndrome Coronavirus 2 (COVID-19) (Knowledge Update).

[16] Use BCCoS (2020). COVID-19: information for opioid agonist treatment prescribers and pharmacists.

[17] British Columbia Centre for Substance Use: Risk mitigation in the context of dual public health emergencies. Interim Clinical Guidance. Retrieved from https://www.bccsu.ca/wp-content/uploads/2020/04/Risk-Mitigation-in-the-Context-of-Dual-Public-Health-Emergencies-v1.5.pdf.

[18] The Federal Council of the Swiss Government. Coronavirus: The Federal Council adapts the criteria for the delivery of medical heroin (Berne; 2020. Contract No.: November 18). Retrieved from: https://www.admin.ch/gov/fr/accueil/documentation/communiques.msg-id-80511.html.

[19] Ferri M, Davoli M, Perucci CA (2011). Heroin maintenance for chronic heroin-dependent individuals. Cochrane Database Syst Rev.

[20] Oviedo-Joekes E, Guh D, Brissette S, Marchand K, MacDonald S, Lock K (2016). Hydromorphone compared with diacetylmorphine for long-term opioid dependence: a randomized clinical trial. JAMA Psychiatry.

[21] Mead N, Bower P (2000). Patient-centredness: a conceptual framework and review of the empirical literature. Soc Sci Med.

[22] Scholl I, Zill JM, Harter M, Dirmaier J (2014). An integrative model of patient-centeredness - a systematic review and concept analysis. PLoS One.

[23] Marchand K, Beaumont S, Westfall J, MacDonald S, Harrison S, Marsh DC (2019). Conceptualizing patient-centered care for substance use disorder treatment: findings from a systematic scoping review. Subst Abuse Treat Prev Policy.

[24] Park SE, Grogan CM, Mosley JE, Humphreys K, Pollack HA, Friedmann PD (2020). Correlates of patient-centered care practices at U.S. substance use disorder clinics. Psychiatr Serv.

[25] Merrill JO (2002). Policy progress for physician treatment of opiate addiction. J Gen Intern Med.

[26] Canada H (2020). Subsection 56(1) class exemption for patients, practitioners and pharmacists prescribing and providing controlled substances in Canada during the coronavirus pandemic.

[27] BC Ministry of Health OotPHO (2020). Registered nurse and registered psychiatric nurse public health pharmacotherapy.

[28] Jaffe JH, O'Keeffe C (2003). From morphine clinics to buprenorphine: regulating opioid agonist treatment of addiction in the United States. Drug Alcohol Depend.

[29] Reimer J, Wright N, Somaini L, Roncero C, Maremmani I, McKeganey N (2016). The impact of misuse and diversion of opioid substitution treatment medicines: evidence review and expert consensus. Eur Addict Res.

[30] Wright N, D'Agnone O, Krajci P, Littlewood R, Alho H, Reimer J (2016). Addressing misuse and diversion of opioid substitution medication: guidance based on systematic evidence review and real-world experience. J Public Health (Oxf).

[31] Johnson B, Richert T (2015). Diversion of methadone and buprenorphine from opioid substitution treatment: patients who regularly sell or share their medication. J Addict Dis.

[32] Johnson B, Richert T (2015). Diversion of methadone and buprenorphine from opioid substitution treatment: the importance of patients' attitudes and norms. J Subst Abus Treat.

[33] Reddon H, Ho J, DeBeck K, Milloy MJ, Liu Y, Dong H (2018). Increasing diversion of methadone in Vancouver, Canada, 2005-2015. J Subst Abus Treat.

[34] Marchand K, Foreman J, MacDonald S, Harrison S, Schechter MT, Oviedo-Joekes E (2020). Building healthcare provider relationships for patient-centered care: a qualitative study of the experiences of people receiving injectable opioid agonist treatment. Subst Abuse Treat Prev Policy..

[35] Swiss Society of Addiction Medicine. Medical recommendations for opioid agonist therapy (OAT) for opioid dependence syndrome - 2020. Bern: Switzerland; 2020.

